# Supplementary material for: Are carbon emissions trading and green financial instruments synergistic? -Comprehensive quantitative research based on content analysis
Source: PLoS One. 2024 Mar 7;19(3):e0298601. doi: 10.1371/journal.pone.0298601 (PMC10919601; doi:10.1371/journal.pone.0298601)
Supplement: S1 Data — (ZIP) [file pone.0298601.s001.zip › Data/地级市Z_F工作报告.zip!/╕ú╜¿/╞╬╠∩╩╨/└┤╫╘╣1⁄2╓┌║┼í╛┬φ┐╦╩2╛▌═°í┐_2011.docx]

各位代表：

现在，我代表莆田市人民政府向大会报告工作，请予审议，并请市政协各位委员和其他列席人员提出意见。

2010年及“十一五”工作回顾

2010年，在中共莆田市委的正确领导下，全市上下坚持以科学发展观为指导，深入学习贯彻胡锦涛总书记在福建考察时的重要讲话精神，全面落实省委八届九次、十次全会精神和省委、省政府“五大战役”的决策部署，大力实施“以港兴市、工业强市”发展战略，打好“六大战役”，实现“五大突破”，落实“两大保障”，顺利完成了市五届人大五次会议批准的各项目标任务。初步预计，全市生产总值815亿元，增长15%。全社会固定资产投资480亿元，增长32%。财政总收入78亿元，增长24%，其中地方级财政收入47亿元，增长24%。社会消费品零售总额280亿元（新口径），增长18%。城镇居民人均可支配收入和农民人均纯收入分别增长10%和11%。农林牧渔业总产值145亿元，增长4%。工业总产值1330亿元，增长22%。按可比口径，合同利用外资7.8亿美元，增长117%，实际到资7.7亿美元，增长17%。外贸进出口总额32.4亿美元，增长38%，其中出口21.6亿美元，增长30%。重点项目建设有力推进，303个重点项目实现年度投资301.7亿元。争取中央增投项目103个、资金4亿元。

农村经济平稳发展。强农惠农政策全面落实，市级以上财政投入“三农”资金5.1亿元，增长78.1%。粮食生产保持稳定，播种85万亩，总产32.5万吨。农业产业化水平不断提升，120家市级以上农业产业化龙头企业销售收入130亿元，增长22%，带动农户创收27.9亿元。新农村建设持续推进，100个中心村建设水平得到提升。挂钩帮扶重点贫困村，落实帮扶资金1986万元。基本完成饮水安全村村通工程。除险加固病险水库22处，加固海堤29.3公里，整治农村河道47.8公里，新增节水灌溉5万亩，治理水土流失0.6万亩。新增农村公路266公里、农村户用沼气池5660口。建成石城一级渔港。农业生产综合机械化水平超过35%。“造福工程”、“阳光工程”分别完成省定3700人、3900人任务。金钟大型水库建成蓄水，引水配套工程开工建设。木兰溪下游防洪二期霞林段工程基本建成，三期荔涵段工程完成年度任务。

工业经济有力提升。规模工业企业总数达1358家，规模工业产值1225亿元，增长25%。工业经济效益综合指数达到230%，工业品产销率98%，实现利润总额43亿元，增长35%。轻重工业结构进一步优化，轻重工业比例为69.8:30.2，重工业比重首次突破30%。鞍钢冷轧、国投湄洲湾石门澳产业园基础设施工程、LNG3-4#储罐、天喔（福建）食品有限公司三期茶饮料、闽中食品工业园、仙游工艺产业园等项目建设加快。亿发工贸园一期、中科万邦LED项目建成投产。赛得利差别化纤维、福人木业、南日风电四期、精细胶粉开工建设。丁基橡胶、众和股份扩建工程前期工作有效推进。

第三产业不断增强。城乡消费持续增长，家电下乡、以旧换新工作成效显著，累计实现销售21.6万台、4.6亿元，补贴金额5512万元。传统商贸业持续发展，涵江国际商贸城一期等项目投入运营，福建海峡国际商贸城、红星美凯龙家居城开工建设。旅游业加快发展，全年累计接待游客880.7万人次，旅游总收入75.2亿元，分别增长22.5%和20%。住宿餐饮业整体接待能力显著提升，17家星级酒店建成营业。房地产业平稳发展，房地产开发完成投资92亿元，商品房销售面积165万平方米，销售额81亿元，分别增长45%、20%和40%。担保体系建设继续加强，新设立融资性担保公司11家、注册资本金8.7亿元，融资担保11.7亿元。金融服务业加快发展，年末全市金融机构人民币存贷款余额分别为730亿元和625亿元，分别增长19.8%和25.7%，莆田农村商业银行顺利组建，民生银行落户莆田。

交通建设有效突破。交通基础设施完成投资100.8亿元, 增长147%。港口码头加快建设，湄洲湾深水航道二期东吴航道工程基本完成，东吴作业区1-6#泊位、东1-2#泊位建设加快，莆头作业区1-2#泊位主体工程开工建设。福厦铁路全线建成通车，向莆铁路莆田段、湄洲湾港口铁路支线有力推进。沈海线扩建工程莆田段基本完成，莆永高速公路莆田段建设加快，沈海复线莆田段开工建设，湄洲湾至重庆高速公路莆田段前期工作有效推进。省道202线黄文公路拓宽工程、省道201线仙游段、仙港大道等项目建成通车，城港大道二期、石城疏港路等项目抓紧建设，省道201线太湖至东沙段、莆兴大道二期工程等项目相继开工，罗屿疏港公路等项目前期工作基本完成。莆田机场前期工作有序推进。

宜居城市加快建设。完成城市总体规划、土地利用总体规划、宜居莆田建设规划、湄洲岛总体规划、木兰溪两岸城市设计及城市综合交通、生态绿心保护、城市抗震减灾、南北洋防洪排涝等专项规划编制工作。建筑业快速发展，总产值首次突破100亿元。城乡路网不断完善，完成荔城大道等4条道路“白改黑”工程，东海路等省运会配套道路竣工通车。完成市体育中心场馆群、莆仙大剧院等市区标志性建筑物工程及园林绿化、夜景工程建设任务，市容市貌明显改观。小城镇综合改革建设试点工作扎实推进，完成江口、西天尾2个省级试点镇和7个市级试点镇的总规编制工作。节能减排取得突破，全年共投入4.9亿元。东圳、外度水源保护区畜禽养殖污染治理强力推进，全市共拆除畜禽养殖场6544个。植树造林6.2万亩，森林覆盖率达58%。新建、扩建污水处理厂7座，新增污水处理能力16.5万吨/日，完成配套管网建设296公里，污水处理率达到80%以上。大湖垃圾处理厂二期扩建、垃圾焚烧发电厂和垃圾转运站建设进展顺利。单位生产总值能耗下降 3％。二氧化硫排放量削减6000吨，化学需氧量排放削减2600吨。加强耕地保护，超额完成省下达补充耕地和土地整理任务，连续十一年实现耕地占补平衡。

社会民生持续改善。科技创新能力不断增强，高新技术产品增加值实现57亿元，增长30%。教育事业优先发展，完成中小学校安工程校舍改造33.6万平方米，市特殊学校建成投入使用，启动莆田电大、莆田职校、仙游师范学校迁建工作，农村寄宿制学校建设工程进展顺利。改造、新建乡镇文化站33个、农家书屋185个，莆仙戏下乡演出活动成效明显。第三次全国文物普查和“妈祖信俗”等非物质文化遗产保护工作有序展开。成功举办福建省第十四届运动会和第八届老年人体育健身大会，全民健身活动蓬勃开展。市、区疾控中心投入使用，莆田学院附属医院新区、市第一医院门诊大楼、市妇幼保健院等项目有序推进。市第一医院、莆田学院附属医院晋级三级甲等综合医院。新农合参合率98.4%，（上接Ａ１版）筹资水平提高到每人每年150元。顺利开展第六次全国人口普查工作。人口和计划生育工作扎实推进，人口出生率、自然增长率、出生政策符合率均完成省下达的控制目标。城镇新增就业2.2万人，城镇登记失业率为2.56%，低于目标控制数0.44个百分点。城乡居民最低生活保障制度进一步完善。社会福利事业持续推进，农村养老保险试点工作稳步推进，20个居家养老试点单位和4所养老院基本建成。新建廉租住房1390套，新增租赁补贴639户，建设经济适用住房1000套，回迁安置2730户。10件为民办实事项目基本完成。民族宗教工作有效开展。防灾减灾工作顺利推进。重大安全隐患排查和专项整治全面展开。食品药品监督管理有效加强。“平安莆田”建设扎实推进，有效防范、严厉打击违法犯罪行为，社会和谐稳定。

一年来，各级政府加快职能转变，加强法制建设，积极推进简政放权制度改革，提高行政审批效率，三向并联审批带动提速，按期办结率98.9%，即办率19.3%。强化行政监察、审计和工作督查，全面落实廉政建设责任制，政府自身建设迈出新步伐。

各位代表，过去的一年，湄洲湾港口城市建设取得新成效。这是市委统揽全局、科学决策的结果；是市人大、市政协有效监督和大力支持的结果；是全市干部群众团结奋斗、努力拼搏的结果。在此，我代表市人民政府，向全市广大干部群众，向人大代表、政协委员和离退休老同志，向各民主党派、工商联、各人民团体、无党派人士和社会各界人士，向中央及省驻莆单位，向驻莆部队指战员、武警官兵和公安干警，向所有支持和关心莆田发展的港澳台同胞和海内外朋友，表示崇高的敬意和衷心的感谢！

各位代表，2010年工作任务的完成，标志着“十一五”规划胜利实现，经济社会发展迈上一个新台阶。

经济持续快速发展，综合实力明显增强。预计五年间全市生产总值、财政总收入、地方级收入年均增长15.2%、21%、25%。人均生产总值突破2.8万元。社会消费品零售总额年均增长17%。累计完成全社会固定资产投资超过1500亿元，是“十五”时期的4.4倍。现代农业发展壮大，农林牧渔业总产值年均增长5.3%。工业总产值突破1300亿元，十大产业集群逐步壮大。与央企项目对接效果明显，中海油、中铁、国投等央企项目落户莆田，累计完成投资700多亿元，福建LNG项目秀屿接收站、莆田燃气电厂等一批临港工业项目建成投产。服务业增加值年均增长14.1％。新增中国名牌产品3个，中国驰名商标11个，国家出口免验产品2个。

城乡面貌显著变化，城市形象稳步提升。完成交通运输、仓储、邮政业投资332亿元，是“十五”时期的9.6倍。城市建成区面积由“十五”末的43.8平方公里扩大到66.8平方公里，城镇化水平达47.5%。加快新农村建设，累计完成38个集镇、600个村总体规划和332个村镇住宅片区详细规划。实施农村家园清洁行动和农村饮水安全村村通工程，农村生活垃圾无害化处理率达80%以上，全市水环境明显改善，“十一五”节能减排目标基本完成。

改革开放持续深入，发展活力不断增强。农村综合改革稳步推进，流转农用地36.8万亩，完成522个村的商品林和498个村生态公益林管护机制改革任务。深化行政管理体制改革，建立重大事项集体决策机制、经济发展激励机制、重大项目推进机制、环境优化促进机制。国企改革基本完成，雪津啤酒国有股权成功转让，整合组建港务、建工、水务、国投、城投、交投、高速、湄控八大集团（公司）。推进企业改制上市工作，众和股份、闽中有机食品成功上市。加大招商引资力度，成功举办莆商大会、香港经贸周、艺博会、木洽会、妈祖文化旅游节、南少林武术文化节等活动。预计五年累计实际利用外资（按可比口径）30.3亿美元，是“十五”时期的2.5倍；全市外贸累计进出口总额117亿美元，年均增长15.2%。莆台交往加强，秀屿港（含湄洲岛）被列为国家首批两岸直接“三通”口岸，湄洲与马祖首次通航。支持彭州濛阳镇灾后重建工作圆满完成，对口支援工作深入开展。

社会事业全面发展，人民生活显著改善。实施科教兴市战略，蝉联“全国科技进步先进城市”三连冠。全面实现“双高普九”。妈祖信俗申报世遗成功，莆仙戏列入国家非物质文化遗产目录。有效应对甲型流感等重大疫情。社区卫生服务中心覆盖率达100%。认真落实人口与计划生育、国土资源、环境保护基本国策，连续五年完成省下达指标。城镇居民人均可支配收入和农民人均纯收入年均分别增长11.5%和11%。城镇累计新增就业人员11.1万人，实现下岗再就业11.6万人，转移农村劳动力19.2万人，城镇职工登记失业率五年均控制在2.6%以下。城乡低保实现应保尽保，以社会保险、社会救助、社会福利为基础的社会保障体系逐步完善。连续三届获得“全国双拥模范城”称号。

回顾过去的五年，我们十分清醒地看到，全市经济社会发展中还存在不少困难和问题。主要是：经济总量仍然较小，产业结构不尽合理；港口优势尚未充分发挥，产业集聚能力不强；现代服务业发展较慢，高新技术产业比重不高，土地、资金、人才、生态环境等制约趋紧；安全生产、防灾减灾措施在基层尚未全面落实；违法用地、违章建设问题依然存在，城市建设和管理水平有待提升；信访问题还比较突出，社会治安状况不尽人意，社会安定稳定存在不确定因素等等；政府工作人员的服务意识、创新意识、执法意识仍有待增强。对此，我们要高度重视，采取有效措施，认真加以解决。

坚持以科学发展观统领“十二五”时期发展

“十二五”时期，是我市加快发展的关键时期。我们要准确把握国内外、省内外形势，加快转变经济发展方式，全力推动我市科学发展、跨越发展。

“十二五”时期全市经济社会发展的指导思想是：高举中国特色社会主义伟大旗帜，以邓小平理论和“三个代表”重要思想为指导，深入贯彻落实科学发展观，按照中央“五个坚持”和省委“先行先试、加快转变、民生优先、党建科学”的基本要求，以科学发展、跨越发展为主题，以加快转变经济发展方式为主线，以改善民生为立足点，更好地融入福建和海西发展大局，坚持用发展凝聚人心、用发展提升形象、用发展改善民生、用发展考核干部，大力实施“以港兴市、工业强市”发展战略，全面推进湄洲湾港口城市和世界妈祖文化中心建设，凝心聚力、增创优势，抢抓机遇、加快转变，深化改革、扩大开放，改善民生、促进和谐，推动跨越发展、建设宜居港城，为提前三年建成全面小康社会奠定决定性基础。

“十二五”时期经济社会发展的总体目标是：跨越发展、宜居港城。

———经济实现跨越发展。全市生产总值年均增长16%以上，到2015年确保1800亿元、力争达到2000亿元，比2010年翻一番以上；人均生产总值6.6万元以上，力争达到全省平均水平；财政总收入年均增长18%以上，到2015年确保180亿元、力争达到200亿元，比2010年翻一番以上；全社会固定资产投资年均增长25%，五年累计5000亿元；社会消费品零售总额年均增长18%；外贸进出口年均增长10%；五年累计实际利用外资40亿美元（按可比口径）。

———结构调整不断优化。经济社会发展转型取得明显进展，先进制造业和服务业贡献率明显提升，三次产业在更高层次上协调发展，基本建成现代产业体系，发展质量和效益进一步提高，发展活力和后劲进一步增强。到2015年，三次产业增加值比重调整为7：55：38；城镇化率提高到55%，力争城乡一体化走在全省前列；高新技术产业增加值占GDP比重力争达15%；知识产权拥有量提高，研发经费支出占GDP比重达1%以上，力争达到全省平均水平；形成一批拥有自主知识产权和知名品牌、竞争力强的大企业大集团；基本建成以临港工业为主导的海峡西岸先进制造业基地，我市在全省的地位和作用更加凸显。

———人民生活更加殷实。城乡居民收入大幅度提高，基本形成合理的收入分配结构，居住、交通、生态等生活环境有较大改善，人民群众的幸福指数明显提升。城镇居民人均可支配收入年均增长11%，农民人均纯收入年均增长10%以上。人口自然增长率年均控制在8‰以内，2015年末全市常住人口300万人。城镇登记失业率控制在4%以内；五年城镇新增就业人数10万人。价格总水平保持基本稳定。

———公共服务逐步完善。覆盖城乡居民的社会保障体系基本形成，公共服务均等化水平明显提高。到2015年，财政教育支出占财政一般预算支出比重达37%；九年义务教育巩固率达97.5%；主要劳动年龄人口平均受教育年限10.5年；高中阶段教育毛入学率达90%；城镇基本医疗保险参保率95%以上；新型农村合作医疗参合率达98%以上；新型农村社会养老保险参保率100%；城镇保障性住房建设套数完成省下达任务；千人均医疗机构床位数3.4张；千人均医生1.7人。

———生态环境优美宜居。生态市建设取得重要阶段性成果，经济发展、人口布局和资源环境承载力更趋协调，基本建成生态效益型经济体系，基本建成优美舒适的人居环境，达到“较宜居城市”标准。到2015年，城市（含县城）垃圾无害化处理率95%以上；城市（含县城）污水处理率80%以上；耕地保有量不低于108.69万亩；非化石能源占一次能源消费比重25%；单位工业增加值用水量、单位GDP二氧化碳排放降低、单位GDP能源消耗、主要污染物排放减少等均完成省下达任务；森林蓄积量800万立方米；森林覆盖率59.8%。

实现以上发展目标，要突出科学发展、跨越发展；突出转变方式、调整结构；突出以港兴市、工业强市；突出先行先试、改革开放；突出统筹协调、改善民生。要着力优化区域发展布局，形成“一核两翼三基地”的发展格局；着力构建现代产业体系，加快建设海峡西岸先进制造业和能源基地；着力实施扩大内需战略，形成内需拉动强劲的经济增长趋势；着力统筹城乡发展，加快建设滨海宜居城市；着力完善基础设施建设，打造东南沿海区域性交通枢纽；着力推进全方位开放，大力发展开放型经济；着力增创莆台合作发展新优势，构筑两岸人民交流合作的前沿平台；着力提高可持续发展水平，构建资源节约型、环境友好型和人口均衡型社会；着力保障和改善民生，努力构建和谐社会；着力推进改革攻坚步伐，建立更具活力的体制机制。

努力为“十二五”发展起好步开好局

2011年是“十二五”发展的开局之年，要进一步开拓创新、扎实工作，继续实施“五大战役”，以优异的成绩迎接建党90周年。今年经济社会发展的主要目标是：力争实现“超千争百促跨越”。全市生产总值增长16%，预期970亿元，力争突破千亿元；规模以上工业总产值增长25%，预期1560亿元；全社会固定资产投资增长35%，预期650亿元，力争突破800亿元；社会消费品零售总额增长18%，预期330亿元；外贸进出口总额增长13%，预期36.6亿美元，其中出口23.8亿美元，增长10%，进口12.8亿美元，增长18.5%；实际利用外资（按可比口径）8亿美元，增长4%；财政总收入95亿元，增长22%，力争突破百亿元；城镇居民人均可支配收入和农民人均纯收入分别增长12%以上；城镇登记失业率控制在3%以内；居民消费价格指数涨幅控制在3%左右；人口自然增长率控制在7.5‰以内；耕地保有量、单位生产总值能耗、主要污染物排放等约束性指标均完成省下达任务。

实现今年经济社会发展目标，必须抓好八个方面工作：

一、夯实三农基础，促进农村经济稳步发展

促进农民增收。落实各项强农惠农政策，足额兑现各类补贴资金。实施促进农民增收十大工程。支持发展粮食产业，促进种粮农民增收。搞活土地流转机制，提高土地经营效益。加强农村劳动力素质培训，鼓励农民就业创业。加快发展农民专业合作社，深化农产品流通，提高农业社会化服务水平。

发展现代农业。转变农业发展方式，调整优化农业结构，大力发展水产、水果、蔬菜、林业等优势产业。把农产品加工作为工业大产业来抓，扶持发展农业龙头企业，完善龙头企业与农户的利益联动机制，引导企业发展规模化农产品加工。推进农业标准化示范建设，打造农产品无公害基地。加强重大动植物疫病防控，完善农产品市场准入制，建立健全农产品质量安全检验检测体系，确保农产品质量安全。深化莆台农业合作，建好仙游台湾农民创业园。

推进新农村建设。统筹完善村庄规划，抓好100个中心村建设，培育打造一批新农村建设精品示范村。认真落实20个重点贫困村扶贫帮困工作。完善路网工程，完成安保工程266公里，改造危桥16座。续建木兰溪下游防洪工程，启动木兰溪中上游（仙游段）防洪工程建设。除险加固17座小型水库。基本完成乌溪水库前期工作。继续实施新农村饮水安全工程，力争解决海岛村群众饮水安全问题。继续建设东圳大型灌区和萩芦中型灌区。实施6片节水示范工程。推进涵江、仙游小型农田水利重点县建设。抓好石城中心渔港等5个渔港建设。深入开展农村家园清洁行动。不断健全农村教育、卫生、文化等设施，发展农村社会事业。

二、建设工业强市，促进工业经济提速增效

培育壮大临港产业。整合“三湾”资源，重点推进铁矿石、木材、油品、LNG和煤炭“五行”基地建设。建成投入使用南日风电四期、后海风电场等风电项目；推进龙源福建总部和平海湾潮汐能发电项目建设。开工建设LNG5-6#储罐、丁基橡胶、中新石化东吴仓储等项目。加快建设湄洲湾LNG产业园、秀屿木材加工区、石门澳产业园、东吴产业基地和仙游精细化工产业基地，确保鞍钢冷轧、冷能空分、赛得利差别化纤维一期等重大项目实现投产。扎实推进大型钢铁基地、新型超超临界火电、LNG大型储备基地、中投釜山石化、国投湄洲湾硅钢等重大项目前期工作。继续推进莆田核电、核工业产业园区前期工作，争取列入国家核电“十二五”规划。依法清理闲置土地和岸线，集约利用港口资源。

提升特色产业。认真落实“2135”产业发展规划，实施传统特色产业技术创新提升工程。促进福晶科技与中科万邦合作开发LED应用照明及上游芯片产品项目。积极扶持三棵树、闽中有机食品、新飞天鞋业、新威电子、连天红、新东亚机械等一批新增长点龙头企业扩产增值，加快建设宝泉古典工艺集中区、亿发工贸园、仙游循环经济示范基地等项目，开工建设天下农庄粮油深加工、华港制油、海峡纺织工业园、海山机械、万鑫金属制品产业园（迁建）等项目。

构筑自主创新高地。实施“企业研发投入资助计划”，重点扶持一批工程研究中心、工程实验室和企业技术中心，提升企业自主创新能力。加强院地合作，推进产学研结合。鼓励企业参与制定国家、行业标准。继续实施品牌带动，扩大品牌效应，推行敏捷制造，提高产业竞争力，争取新增省级企业技术中心2家、行业性和区域性企业技术创新公共服务平台1个，推进企业开发新产品200项以上，申请省新产品鉴定5项以上，新创国家级品牌3个、省级品牌30个。

加快发展民营经济。继续实施“民资回归”工程。优化民营经济发展环境，切实形成服务民营企业发展的绿色通道。从具体企业入手，做好大型民营企业制造业项目洽谈工作，签约一批项目。

促进企业改制上市。加大政策扶持力度，鼓励企业改制上市。积极争取三棵树、保兰德两家企业挂牌上市。主动帮助双驰鞋业、才子服饰等已改制企业加快上市步伐。大力培植华丰鞋业、华林蔬菜、金利莱斯服饰、集友框业等拟上市后备企业，加快完成股份制改造。

三、扩大服务需求，促进第三产业蓬勃发展

培育现代物流业。加强运输市场监管，鼓励交通运输企业开拓专业化、社会化物流服务。积极发展第三方物流，筹建成立福建凤凰国际航运有限公司，培育一批现代物流龙头企业。加快建设湄洲湾配煤基地、涵江临港物流园区、铁路港湾站物流园区、黄石商贸物流园区、秀屿综合物流园。开工建设东吴60万立方米商业油库储备基地、罗屿铁矿石中转基地、东吴物流园区等项目。加快推进盘屿250万立方米商业储备油基地等项目前期工作。

提升商贸服务业。开拓城乡市场，扩大居民消费，构建大型消费平台，继续引进国内外知名大型商业零售企业，加快建设海峡国际商贸城、红星美凯龙、鞋服城、珠宝城、古典家具城等大型商场、专业市场，推进钢贸城、汽贸城、家居建材城等项目前期工作。规范房地产市场秩序，扩大廉租房、公共租赁房、经济适用房供应量，积极拓展二手房和住房租赁市场，大力发展健康住宅、绿色环保住宅和节能省地型住宅，促进多层次的住房消费。大力发展便民商业和社区生活服务业，完善社区商业网点，加快集贸市场升级改造，扎实推进城市便民店建设，提升城市消费水平。继续实施万村千乡市场工程，推进“菜篮子”工程和“家电下乡”活动，促进农村消费。

发展新兴服务业。大力发展商务服务业，推动总部经济发展，加快建设总部基地和总部商务区，有效吸引跨国公司和国内知名大型企业来我市设立地区总部、研发设计中心、采购营销中心和结算中心。大力发展文化创意产业，推进海西文化创意产业园、妈祖文化影视城等项目前期工作，规划建设工艺美术创意产业基地。整合传媒业经营性资源，优化提升报业、广电、印刷出版等文化产业。积极培育文化娱乐、电子通信、教育培训、体育健身、休闲旅游、家政服务等生活需求型服务业，统筹推进信息、外包、中介、广告、策划、大型会展等商务分包型服务业。

拓展旅游经济。加大湄洲岛创建5A级旅游景区力度，完善景区基础配套和服务接待设施，申报设立妈祖文化旅游综合试验区、海峡两岸文化交流合作示范区和妈祖文化生态保护实验区。统筹开发大九鲤湖景区，带动常太现代农业观光园、麦斜岩、菜溪岩等景区连片开发建设。积极推进海峡、滨海、文化、生态、红色、乡村休闲、工艺旅游。加强旅游区域合作，促进莆台旅游合作，深化闽东北五市的旅游协作机制，主动融入海西大旅游圈。

四、实施项目带动，促进投资拉动强劲有力

推进重点项目建设。着力抓好省级、市级在建、预备和前期重点项目，强力推进百个重大攻坚项目。加强与央企、国内外知名企业的合作。全程跟踪服务项目，强化用地、用海、资金、环评等建设要素的保障力度。

优化项目结构。认真对接国家、省产业调整和振兴实施方案，大力引进加工制造、自主创新、环境友好、高新技术等类型的重点项目，加大对临港工业建设、产业结构升级、城乡统筹发展、社会事业进步、生态环境保护等领域的投资力度，进一步优化投资结构。

构建综合交通体系。加快建设湄洲湾航道拓宽三期工程及东吴作业区东1-2#、秀屿作业区2-3#、莆头作业区1-2#等深水泊位码头建设。续建向莆铁路莆田段和湄洲湾港口铁路支线，建设福厦铁路枫亭客运站，全力配合宁漳城际轨道、仙龙铁路、宁漳客货运专线的前期工作。继续推进莆田机场前期工作。建成沈海高速公路莆田段扩建工程，续建莆永高速公路莆田段、沈海高速公路复线莆田段，开工建设湄洲湾至重庆高速公路莆田段。加快省道201线莆田段、202线莆田段、城港大道二期、石城疏港路、秀屿疏港路、涵港路等疏港道路建设。优先发展城市公共交通，逐步建立城乡统筹的公交体系。

五、统筹城乡发展，促进宜居城市加快建设

完善城市基础设施。建设秀港路、仙游二环路、涵江环城南路、木兰大道、物流1#、2#路等市政道路，打通学园路北段、八二一街顶墩段、胜利路等断头路，实施荔园路等城市主干道“白改黑”工程。继续实施城市主干道电缆下地工程，新增地下缆线18公里。促进供水资源整合，抓紧完善燃气管道供应系统，建设垃圾处理、转运设施和污水收集配套管网，建成36座符合设置规划标准的城市公厕和一批公共停车场。建设城市规划展示馆，进一步加强城市管理，提升城市形象。

创建国家园林城市。围绕“五山簇拥、四水相依、三湾环绕、二岛添辉、一心为源”的宜居城市建设格局，建成大型城市综合性公园1座，创建绿色乡镇12个，新增立体绿化150处。重点实施荔港大道、荔涵大道、城港大道、迎宾大道、仙港大道、东园路、荔园路二期等城市主干道绿化。启动囊山、九华山、天马山、壶公山、大蜚山等公园规划工作，建设绶溪公园一期、土海湿地公园一期、东岩山公园，改造凤凰山公园。建成城市林荫景观道。建设木兰溪北洋水系荔枝林休闲景观带。

打造宜居城市综合体。严格控制和保护木兰溪两岸土地资源。加快实施木兰溪两岸市政配套基础设施工程，加快推进玉湖片区、木兰金融财富中心、木兰国际广场、莆田火车站站前片区、荔城新区、磐龙山庄、萩芦山庄、泗华滨溪小区、总部经济办公区、樟林水乡等城市综合体和棚户区改造基础设施工程建设，促进新城形成规模，推动旧城完善配套，进一步集聚人气。

加快推进小城镇建设。抓好江口镇、西天尾镇2个省级以及7个市级小城镇综合改革建设试点工作，扩大试点镇范围。完善道路、公交、燃气、供水、污水垃圾处理系统等市政公用基础设施建设，加大文化、教育、卫生、商贸、酒店等服务设施投入，打造特色支柱产业，促进城乡统筹发展。

推进生态文明建设。加强环境质量监管，严格执行规划环评制度。完善矿产资源和水资源的生态补偿机制。深入开展节水型社会建设，大力整治木兰溪、萩芦溪重点流域水环境，扎实推进节能减排工作。全力创建森林城市，继续实施“绿色城市、绿色村镇、绿色通道、绿色屏障”工程，植树造林15万亩，提高生态环境承载能力。

六、深化改革开放，促进发展活力持续增强

推进重点领域改革。加快投融资体制改革，进一步探索和推行BOT、BT等项目融资方式，引导社会资金投向公共基础设施和公共服务领域。拓展公共资源市场化配置工作，规范与完善政府采购、工程、土地、产权等领域的电子化交易。继续推进污水处理、垃圾处理、道路保洁等市场化运作、产业化经营。加快国有企业改革重组步伐，完善以五大产业集团、三大投资性公司为主导的国有资产运营架构，盘活国有存量资产。加强水、电、成品油、药品、医疗服务等重要商品和服务的价格改革。推进政府机构改革，加快事业单位分类改革和人事制度改革。积极构建产业人才高地，营造尊重人才的社会氛围。适时启动行政区划调整。

促进区域协调发展。着力优化区域发展布局，推动形成“一核两翼三基地”的发展格局。集中要素资源全面开发湄洲湾北岸。启动兴化湾南岸规划建设，科学谋划兴化湾南岸产业布局。重点支持仙游加快发展，在政策、项目和融资上给予更大的倾斜，完成金钟水利枢纽扫尾工程，加快引水配套工程建设步伐，做大做强仙游经济开发区、仙游工艺产业园等一批各具特色的工业园区，培育壮大工艺美术、石化等一批产业集群，推动仙游经济奋力崛起。

提升利用外资水平。加大对国际大企业、大公司投资高新技术产业、现代服务业、高端制造业的引导力度，推进金鹰林浆纸一体化、赛得利差别化纤维二期、台湾宏利高世代面板以及力宝、嘉里、信和、佳通、兴隆等集团重大投资项目尽快落地。全力支持鼎盛五金等一批外资企业做好上市筹备工作。吸引外资企业在莆设立地区总部。鼓励台商及境外资本投资我市医疗服务产业。积极参与国内外重大招商展会，重点组织好台湾、新加坡经贸活动周等招商活动，扩大东南亚招商成果。

加强对外交流合作。积极实施“走出去”和出口市场多元化战略，鼓励优势企业开拓海外市场。充分利用自由贸易区的关税优势，深度拓展东盟市场。加强对台文化经贸合作。积极申报莆田台商投资区，主动承接台湾电子、机械、石化等产业转移。推进油品、木材、煤炭、天然气等中转基地建设。继续办好妈祖文化旅游节、艺博会、鞋交会等节会，吸引国内外采购商和有影响力的零售商来莆设点。加快电子口岸建设，进一步提高口岸通关效率。积极参与闽浙赣皖经济协作区、闽东北协作区交流活动，密切与向莆铁路沿线城市合作。

七、加强财经工作，促进经济发展能力保障

培植壮大财源税源。完善财政扶持机制，重点支持科技含量足、带动能力强、税收贡献率高的重大工业项目，大力培育发展建筑、交通等大型企业集团，扩大支柱财源。加快发展第三产业，落实促进餐饮、娱乐、旅游、会展、物流、商贸流通、房地产等服务业发展的政策，发展税源型经济。创新税收征管模式，切实解决票外开、税外流、车船外挂问题。强化税收征管，依法抓好大型税源、重点税种的征收工作，防范和打击偷逃税行为。盘活城市存量资源，加大土地收储力度，强化土地出让金收支管理，增加政府性基金收入。

提高财政管理水平。深化预算制度改革，保法定增长、保民生支出、保重点建设。完善国库集中支付制度，提高财政资金使用效益。创新投资机制，拓宽融资渠道，集中财力办大事。推进依法理财，加强财政和审计监督，严格预算约束，严格控制和压缩一般性预算开支，降低行政成本。

推动金融服务创新。拓展金融服务规模，用活金融服务产品，优化信贷资金投向结构，加大对产业升级、中小企业、节能减排、木兰溪两岸建设、新农村建设等重点领域和薄弱环节的信贷支持。推进债券市场融资，鼓励发展风险投资。争取多家国内外金融机构来莆设点。建立健全融资性担保公司的监管体系，扶持发展中小企业贷款担保机构，成立担保行业协会。创新出口信贷产品，扶持外贸企业发展壮大。加强社会信用体系建设，营造良好金融生态环境。

八、着力社会民生，促进经济社会和谐发展

优先发展教育事业。统筹城乡教育资源，加快城乡义务教育学校标准化建设，创建标准化初中校18所、小学130所。加快中小学校舍安全工程建设，重建中小学校舍50万平方米，全面完成无设防校舍重建任务。全面实施城区中小学扩容工程。推进仙游师范学校、莆田电大和莆田职校新校区建设。大力发展学前教育，加快公办幼儿园建设，鼓励社会力量兴办幼儿园。提升高等教育质量，支持莆田学院和湄洲湾职业技术学院发展。大力发展职业教育，突出培养技能型紧缺人才。重视发展特殊教育，规范发展民办教育，积极发展远程教育、继续教育、社区教育、老年教育、终身教育。加强教师队伍建设，深化教育人事制度改革，做好教师“县管校用”省级试点工作。

提高全民健康水平。加强医疗卫生机构基础设施建设，建成莆田学院附属医院新区，建设市第一医院新门诊大楼和病房大楼，迁（扩）建市妇幼保健院、市皮肤病防治院。扩建市中心血站，兴建义务献血网点。新建改造一批乡镇卫生院、社区卫生服务中心、村卫生（所）室。推进公立医院改革，筹建市级医疗集团，促进民营医院发展。加快实施国家基本药物制度，加强公共卫生服务体系和职业病防治能力建设，强化疾控卫生监督，提高卫生应急处置能力。严格食品药品质量监管，扶持促进中医药事业发展。落实人口与计划生育国策，推进妇幼卫生保健事业，提高出生人口素质。发挥后省运优势，推进全民健身，发展体育产业。

推进文化全面繁荣。继续建设创新型城市，争创全国科技进步先进城市“四连冠”。加快建设博物馆、图书馆、科技馆、艺校等文化设施，全面建成43个乡镇文化站。推进莆仙戏申报“世遗”工作，组建莆仙戏剧院，组织莆仙戏精品创作与演出，做大做强莆仙戏现代文明小戏品牌。推进“妈祖信俗”等非物质文化遗产及文物保护工作，发展妈祖文化产业，培育莆田特色文化市场。完成《莆田市志》总纂。深化精神文明建设，加强社会主义核心价值体系建设，提高人民群众文明素质和创造能力。繁荣发展社会科学、文学艺术、广播影视、新闻出版事业。

扩大就业促进创业。完善就业公共服务体系建设，实施积极的就业扶持政策，重视大中专毕业生就业工作，加大对零就业家庭和困难群体的就业援助力度，新增城镇就业2万人。多渠道开发就业岗位，创建充分就业社区。鼓励自主创业，以创业带动就业。组织各类职业培训，筹建技师学院，建立一条龙创业培训服务模式。实施农村劳动力素质培训工程，转移农村劳动力2.5万人。加强就业信息化建设，推进跨区域劳务合作。

全力办好为民实事。集中力量办好便民商业网点和城市副食品生产基地建设、医疗卫生机构建设、危旧房（棚户区）改造和社会保障房建设、改善城市人居环境、完善公共文化服务设施、教育均衡发展、市民便捷出行工程、无线城市工程、文甲码头游客广场及停车场工程、强化弱势群体帮扶、食品放心工程等关系人民群众切身利益的实事，让人民群众共享发展成果。

完善社会保障体系。规范社会保险管理，扩大各项社会保障政策的覆盖面。全面推行城镇居民、城镇职工基本医疗保险制度，巩固发展新型农村合作医疗制度，健全医疗救助体系。持续建设“金保工程”，提高社会保障能力。推进新型农村社会养老保险工作。深化机关事业单位养老保险制度改革。加快发展慈善、社会福利、老龄和残疾人事业，续建市社会福利中心、市慈康医院和仙游县福利院，推进敬老院建设。加大保障性住房建设力度，新增廉租住房租赁补贴800户，筹集、续建各类保障性和政策性住房6830套。积极调整收入分配格局，实现城乡居民收入增长和经济发展同步、劳动报酬增长和劳动生产率提高同步，切实提高城乡居民收入，让人民生活更加宽裕。

维护社会安定稳定。不断创新“平安莆田”建设机制。推进社会矛盾化解、社会管理创新、公正廉洁执法三项重点工作，严格落实社会治安综合治理责任制，严厉打击“两抢一盗”等各类违法犯罪行为，进一步加强社会治安防控体系建设，确保一方平安。严格落实安全生产责任制，加大安全生产基础设施投入，推进社会消防安全“防火墙”工程，强化监督检查，防止重特大安全事故发生。做好信息化条件下的保密工作，维护国家安全和利益。建立健全防灾减灾体系，提高防灾减灾能力。认真落实党的民族政策，依法加强对宗教场所的整治管理。支持驻莆部队和武警部队工作，加强国防动员和民兵、预备役部队、海防、人防建设，落实双拥优抚安置政策，再创“全国双拥模范城”。继续加强统计、档案、外事、侨务、气象、水文、地震等工作。

加强政府自身建设，构建服务型政府

做好今年各项工作，对政府自身建设提出了新的更高的要求。要坚持一切从人民利益出发，切实转变政府职能，努力建设人民满意的服务型政府。

一、加强依法行政建设。认真落实依法治国基本方略，加强法治政府建设，提高政府公信力。健全重大决策事项专家咨询制度、听证制度和公示制度，提高决策透明度和公众参与度。做好行政执法责任制考核和行政复议工作。自觉接受市人大及其常委会的法律监督，认真执行市人大及其常委会各项决议决定，坚持定期向市人大及其常委会报告工作。主动听取政协和民主党派意见，自觉接受民主监督。切实办好人大代表议案、建议和政协委员提案。支持工、青、妇等群众团体开展工作。开展“六五”普法、法律援助和人民调解工作，完善大调解工作体系，落实信访“路线图”和工作责任制，提高预防处置群体性突发事件能力。推进政务公开，加强电子政务建设，认真实施政府信息公开条例。主动接受新闻媒体的舆论监督，提高政府工作透明度，使权力在阳光下运行。

二、加强行政效能建设。深化行政审批制度改革，加强市、县（区）、镇（街道）服务中心三级平台建设，精简审批，超前服务，提高政府办事效率。规范行政权力运行，深入开展清权确权和政务公开工作。加强绩效管理，抓好行政效能制度建设，建立协调推进机制。强化效能督查和行政问责，重视民生服务监督台的投诉办理，加大对群众关心、社会关注、各方面反映强烈问题的督查力度，严肃查处行政不作为、乱作为和损害群众利益等行为。

三、加强勤政廉政建设。充分理解权为民所赋的深刻含义，始终保持“等不及”、“慢不得”、“坐不住”的紧迫感、危机感和责任感，切实改变机关干部慵懒现象。时刻把解决群众最关心、最直接、最现实的利益问题作为工作的出发点和落脚点，不断强化主动服务的使命感，为基层多办实事，为企业排忧解难，为群众多谋利益。加强惩防体系建设，严格落实党风廉政建设责任制，从源头上杜绝腐败行为。坚持勤俭办一切事业，反对铺张浪费，降低行政成本，把有限的资金用在提高群众生活和增强经济发展的后劲上。加强行政、财政、审计的监督和政府督查工作，坚决纠正损害人民群众利益的不正之风。

各位代表，“十二五”的发展蓝图已经绘就。“以港兴市、工业强市”的发展战略，累积了莆田腾飞的厚重基础。现在，新的发展目标又将引领我们不断夺取新的胜利。莆田，必将奋发崛起。这是一种自豪，也是一种气概。厚积薄发且承接五洲的湄洲湾港，底蕴深厚且远播四海的妈祖文化，勤劳聪慧且勇于创业的莆田人民，永远诠释着、支撑着这种自豪和气概。作为政府，我们将责无旁贷地秉承这份荣耀，与全市人民一道，努力实现“跨越发展、宜居港城”的宏伟目标。

各位代表，港城莆田已迈上新的征程，我们任重道远，肩负着光荣的历史使命，让我们在中共莆田市委的坚强领导下，全面贯彻落实科学发展观，解放思想，同心同德，锐意进取，跨越发展，为全面建设滨海宜居城市而努力奋斗！
